# Contributions of economic growth, terrestrial sinks, and atmospheric transport to the increasing atmospheric CO_2_ concentrations over the Korean Peninsula

**DOI:** 10.1186/s13021-021-00186-3

**Published:** 2021-07-20

**Authors:** Jeongmin Yun, Sujong Jeong

**Affiliations:** Department of Environmental Planning, Graduate School of Environmental Studies, Seoul National University, Seoul, Republic of Korea

## Abstract

**Background:**

Understanding a carbon budget from a national perspective is essential for establishing effective plans to reduce atmospheric CO_2_ growth. The national characteristics of carbon budgets are reflected in atmospheric CO_2_ variations; however, separating regional influences on atmospheric signals is challenging owing to atmospheric CO_2_ transport. Therefore, in this study, we examined the characteristics of atmospheric CO_2_ variations over South and North Korea during 2000–2016 and unveiled the causes of their regional differences in the increasing rate of atmospheric CO_2_ concentrations by utilizing atmospheric transport modeling.

**Results:**

The atmospheric CO_2_ concentration in South Korea is rising by 2.32 ppm year^− 1^, which is more than the globally-averaged increase rate of 2.05 ppm year^− 1^. Atmospheric transport modeling indicates that the increase in domestic fossil energy supply to support manufacturing export-led economic growth leads to an increase of 0.12 ppm year^− 1^ in atmospheric CO_2_ in South Korea. Although enhancements of terrestrial carbon uptake estimated from both inverse modeling and process-based models have decreased atmospheric CO_2_ by up to 0.02 ppm year^− 1^, this decrease is insufficient to offset anthropogenic CO_2_ increases. Meanwhile, atmospheric CO_2_ in North Korea is also increasing by 2.23 ppm year^− 1^, despite a decrease in national CO_2_ emissions close to carbon neutrality. The great increases estimated in both South Korea and North Korea are associated with changes in atmospheric transport, including increasing emitted and transported CO_2_ from China, which have increased the national atmospheric CO_2_ concentrations by 2.23 ppm year^− 1^ and 2.27 ppm year^− 1^, respectively.

**Conclusions:**

This study discovered that economic activity is the determinant of regional differences in increasing atmospheric CO_2_ in the Korea Peninsula. However, from a global perspective, changes in transported CO_2_ are a major driver of rising atmospheric CO_2_ over this region, yielding an increase rate higher than the global mean value. Our findings suggest that accurately separating the contributions of atmospheric transport and regional sources to the increasing atmospheric CO_2_ concentrations is important for developing effective strategies to achieve carbon neutrality at the national level.

## Background

Atmospheric CO_2_ concentrations have risen owing to an increase in anthropogenic CO_2_ emissions, which outweigh natural CO_2_ uptake [1, 2]. To mitigate anthropogenic climate change resulting from rising atmospheric CO_2_ concentrations, countries around the world have pledged efforts to monitor and reduce their CO_2_ emissions through the establishment of the United Nations Framework Convention on Climate Change (UNFCCC) in 1992 [3]. Despite international commitment, over the last few decades, global anthropogenic CO_2_ emissions have increased following the worst-case scenario, in which no action is taken to mitigate carbon emissions [4]. Numerous studies have warned that the increase in global average temperature should be limited to 1.5 ℃ above pre-industrial levels to avoid the risk of irreversible consequences of climate change [5]. Currently, more than 110 nations participating in the UNFCCC have committed to achieving carbon neutrality by 2050 (or 2060), including all East Asian countries, which are responsible for more than half of the global anthropogenic CO_2_ emissions [6, 7]. Individual climate change mitigation policies have been adopted by considering each country’s economic and natural conditions. Therefore, understanding the characteristics of national carbon budgets and their impact on atmospheric CO_2_ changes is essential for establishing effective plans to achieve this goal.

Korea is divided into the Republic of Korea (South Korea) and the Democratic People’s Republic of Korea (North Korea). These countries share similar climate conditions and natural ecosystems considering their geographic proximity, but they have different economic and social histories. In South Korea, the gross domestic product (GDP) has gradually risen since industrialization in the 1970 s, and has increased by 160 % in the last two decades (2000–2016) [8]. Further, forested areas, accounting for 63 % of the country, remained constant with a slight decrease (0.2 %) during 2001–2016 due to sustained national forest management according to remote sensing statistics [9]. Conversely, in North Korea, the GDP increased by 91 % from 2000 to 2016 after an economic collapse in the 1990s; the average GDP ($25 billion) accounted for 2.3 % of South Korea’s GDP ($1053 billion) during the period [10]. The previous economic collapse led to severe deforestation to secure food and fuel, causing the forested areas, accounting for 60 % of the country, to decrease by 1.3 % during 2001–2016 despite international efforts toward forest recovery [9, 11]. The differing changes in the economic and natural ecosystems between these countries can provide insight into the varying carbon dynamics according to human activities and national policies, despite their similar climate and environmental changes.

Spatiotemporal variations in atmospheric CO_2_ concentrations reflect the regional characteristics of carbon sources and sinks [12–14]. Because of the limited spatial coverage of current tower-based surface CO_2_ flux measurements [15, 16], atmospheric CO_2_ has been utilized to diagnose changes in the regional carbon cycle, including both anthropogenic and natural components [17–21]. Long-term measurements show that atmospheric CO_2_ concentrations in East Asia are rising faster than the global average owing to the rapid economic growth of East Asian countries [19, 22]. Satellite measurements have estimated that the column-integrated CO_2_ concentrations in major cities (e.g., Seoul) could be approximately 2 ppm higher than those near non-source (or sink) regions [21, 23]. However, as atmospheric CO_2_ can be significantly affected by atmospheric circulation and regional surface CO_2_ fluxes, it is difficult to monitor changes in the regional carbon cycle solely based on observations. The chemical transport model (CTM) has been used to identify for the drivers of atmospheric CO_2_ variations by separating the influences of regional sources and sinks on CO_2_ variations [19, 24, 25]. Using CTM simulations, Fu et al. [25] estimated that terrestrial CO_2_ flux and fossil fuel CO_2_ emissions account for up to 14 and 17 % of the interannual variations of atmospheric CO_2_ over East Asia, respectively. Yun et al. [19] showed that the observed increasing seasonal difference in atmospheric CO_2_ in South Korea results from enhanced terrestrial carbon uptake. Therefore, CTM simulations could provide help in the interpretation of observed atmospheric signals related to changes in regional carbon budgets.

In this study, we investigated the characteristics of atmospheric CO_2_ variations over South and North Korea during 2000–2016 and identified the causes of their regional differences in the increasing rates of atmospheric CO_2_ concentrations. To accomplish this, we first compared the atmospheric CO_2_ variations in South Korea, North Korea, and the global mean estimated from CTM simulations. Then, changes in the national surface CO_2_ fluxes and energy consumption structure in both countries were examined by analyzing statistical datasets and estimates of process-based modeling and inverse modeling. Finally, we derived the contributions of changes in atmospheric transport, as well as regional anthropogenic and terrestrial CO_2_ fluxes, on increasing atmospheric CO_2_ concentrations over each country based on a set of CTM simulations. The results of the analysis provided a comprehensive understanding of the role of economic activities, terrestrial ecosystems, and atmospheric transport in increasing atmospheric CO_2_ concentrations at the national level.

## Results

### Regional difference in atmospheric CO_2_ trends

**Fig. 1:**
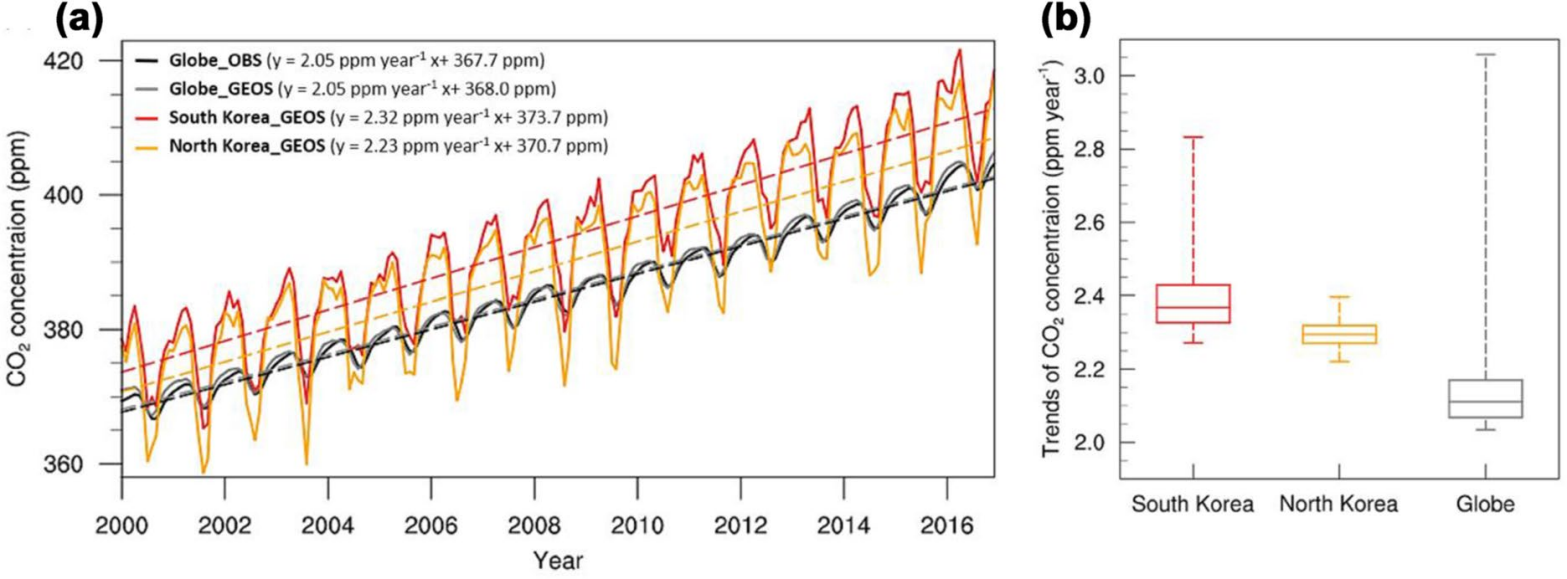
**a** Monthly mean variations of area-averaged atmospheric CO_2_ worldwide (gray), South Korea (red), and North Korea (orange) simulated from a 3-D transport model during 2000–2016. The black line indicates the global monthly mean variations of atmospheric CO_2_ derived from in-situ measurements over the globe. The dotted line represents the linear fit of each monthly variation. **b** Box plots of trends of simulated atmospheric CO_2_ over South Korea, North Korea, and the globe

The CTM modeling estimated that the global mean CO_2_ concentration rose by 2.05 ppm year^− 1^ during 2000–2016, which is consistent with that computed from observations (Fig. 1a). Meanwhile, the simulated rate of atmospheric CO_2_ increase is greater than 2 ppm year^− 1^ in all regions and is more positively skewed, reaching a maximum value of 3 ppm year^− 1^ (Fig. 1b). The increasing rates of atmospheric CO_2_ in South Korea (mean: 2.32 ppm year^− 1^) and North Korea (2.23 ppm year^− 1^) are greater than the global 75 percentile value. These countries also share similar monthly variations in atmospheric CO_2_ (r = 0.98; p < 0.01; two tailed Student’s t-test), especially during the winter when the effect of vegetation activity is negligible (Fig. 1a). However, the variability of the monthly CO_2_ concentration in South Korea was lower than that in North Korea owing to the greater CO_2_ drawdown during the summer in North Korea than in South Korea. Moreover, the maximum increase in atmospheric CO_2_ in South Korea (2.83 ppm year^− 1^) was greater than that in North Korea (2.40 ppm year^− 1^) (Fig. 1b).

### Changes in national surface CO_2_ fluxes

**Fig. 2:**
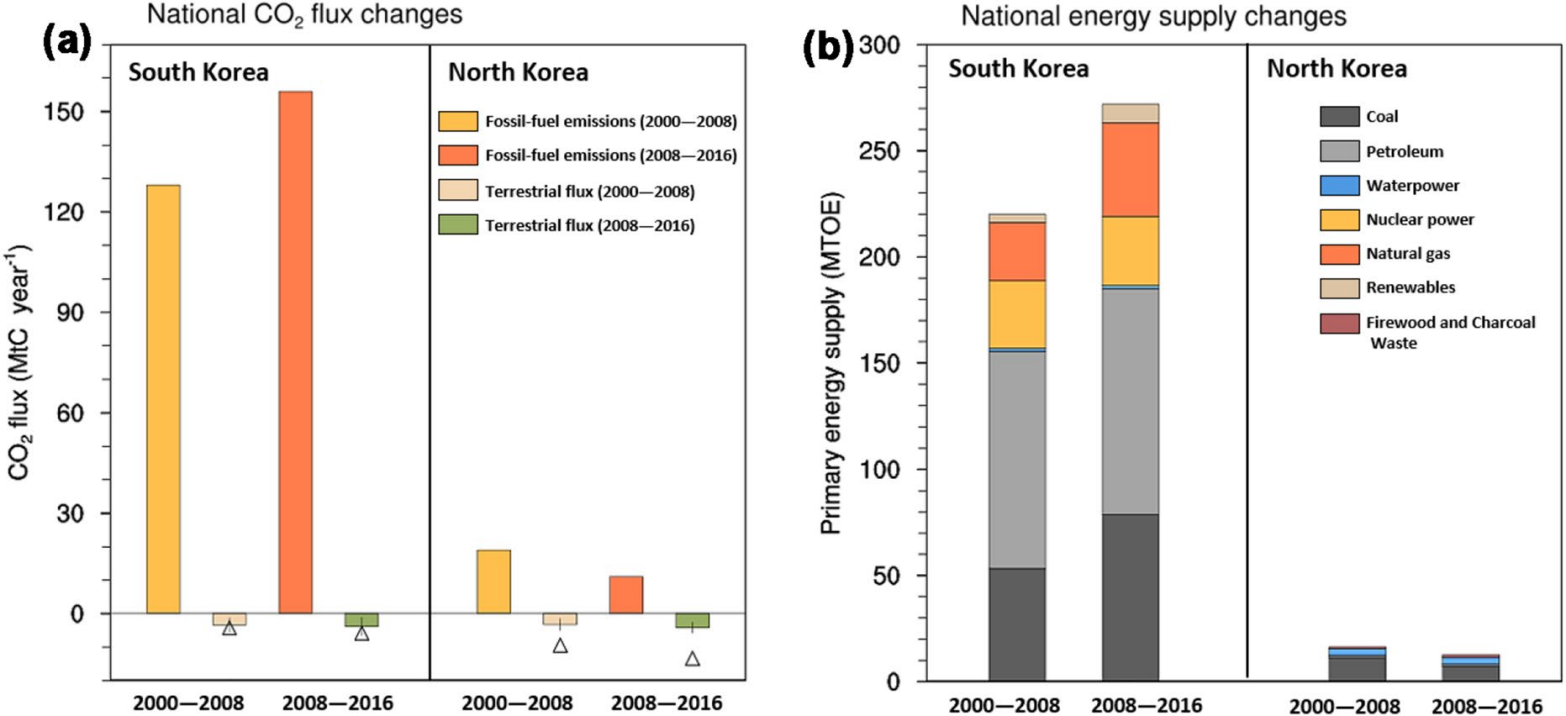
**a** Annually averaged national fossil fuel emissions derived from an inventory and national terrestrial carbon fluxes derived from nine TRENDY multi-model means during 2000–2008 and 2008–2016 in South Korea and North Korea. Positive (negative) values denote CO_2_ emissions (uptake) from land-surface to the atmosphere. Error bars denote one inter-model standard deviation range, and triangles indicate terrestrial CO_2_ flux from CarbonTracker2017. **b** The same as (**a**) but for the primary energy supply of each energy source

The national inventory reported contrasting changes in fossil fuel CO_2_ (FFCO_2_) emissions between the two countries from 2000 to 2016 (Fig. 2a). Specifically, in South Korea, FFCO_2_ emissions increased from 128 MtC year^− 1^ in 2000–2008 to 156 MtC year^− 1^ in 2008–2016. In North Korea, however, FFCO_2_ emissions decreased from 19 MtC year^− 1^ in the initial nine years of the study period to 11 MtC year^− 1^ in the final nine years. These contrasting trends are associated with different histories of changes in energy consumption and structure, as more than 90 % of FFCO_2_ emissions result from energy production in these countries [26]. Economic growth increased energy consumption in South Korea from 220 million tons of oil equivalent (Mtoe) in the initial 9 years to 272 Mtoe in the final 9 years. Further, the ratio of fossil (coal and petroleum) energy consumption decreased by 3 % as a result of increases in natural gas and renewable energy supply, but its magnitude increased by 30 Mtoe between the two periods (Fig. 2b). Conversely, the total energy consumption in North Korea decreased from 16 Mtoe to 13 Mtoe from the initial nine to the final nine years, respectively, as the supply of domestic coal, a major energy source, sharply declined during the study period.

Unlike the FFCO_2_ emissions, both the process-based models (TRENDY) and inverse modeling (CarbonTracker; CT) results estimated that the amount of terrestrial carbon uptake was similar in South and North Korea. These results also estimated increases in terrestrial carbon uptake in both countries during 2000–2016, although the magnitudes differed among the models (Fig. 2a). In particular, the TRENDY models simulated that terrestrial carbon uptake in South Korea increased from 3.4 ± 2.1 MtC year^− 1^ in 2000–2008 to 3.9 ± 2.7 MtC year^− 1^ in 2008–2016, which accounts for 2.5 ± 1.7 % of the mean FFCO_2_ emissions for the period. Similarly, the CT also estimated that the carbon uptake rose from 4.3 MtC year^− 1^ to 5.9 MtC year^− 1^ from the initial nine years to the final nine years, respectively, within an inter-model standard deviation range of the TRENDY models, suggesting that the terrestrial carbon flux in South Korea is well constrained. Similarly, the TRENDY models simulated that the terrestrial carbon uptake in North Korea increased from 3.3 ± 1.8 MtC year^− 1^ in the initial nine years to 4.2 ± 1.6 MtC year^− 1^ in the final nine years of the study period, accounting for 38 % ± 15 % of the mean FFCO_2_ emissions during this period. Further, the CT estimated that the carbon uptake enhanced from 9.4 MtC year^− 1^ to 13.3 MtC year^− 1^ between these periods, which was beyond the standard deviation range of the TRENDY models. This notable discrepancy may have resulted from the absence of atmospheric measurements for constraining the terrestrial carbon flux in North Korea. Overall, these results indicate that South Korea continues to deviate further from achieving carbon neutrality, while North Korea has almost achieved carbon neutrality, even though there exist large uncertainties in the terrestrial carbon flux in North Korea.

### Causes of regional difference in atmospheric CO_2_ trends

**Fig. 3:**
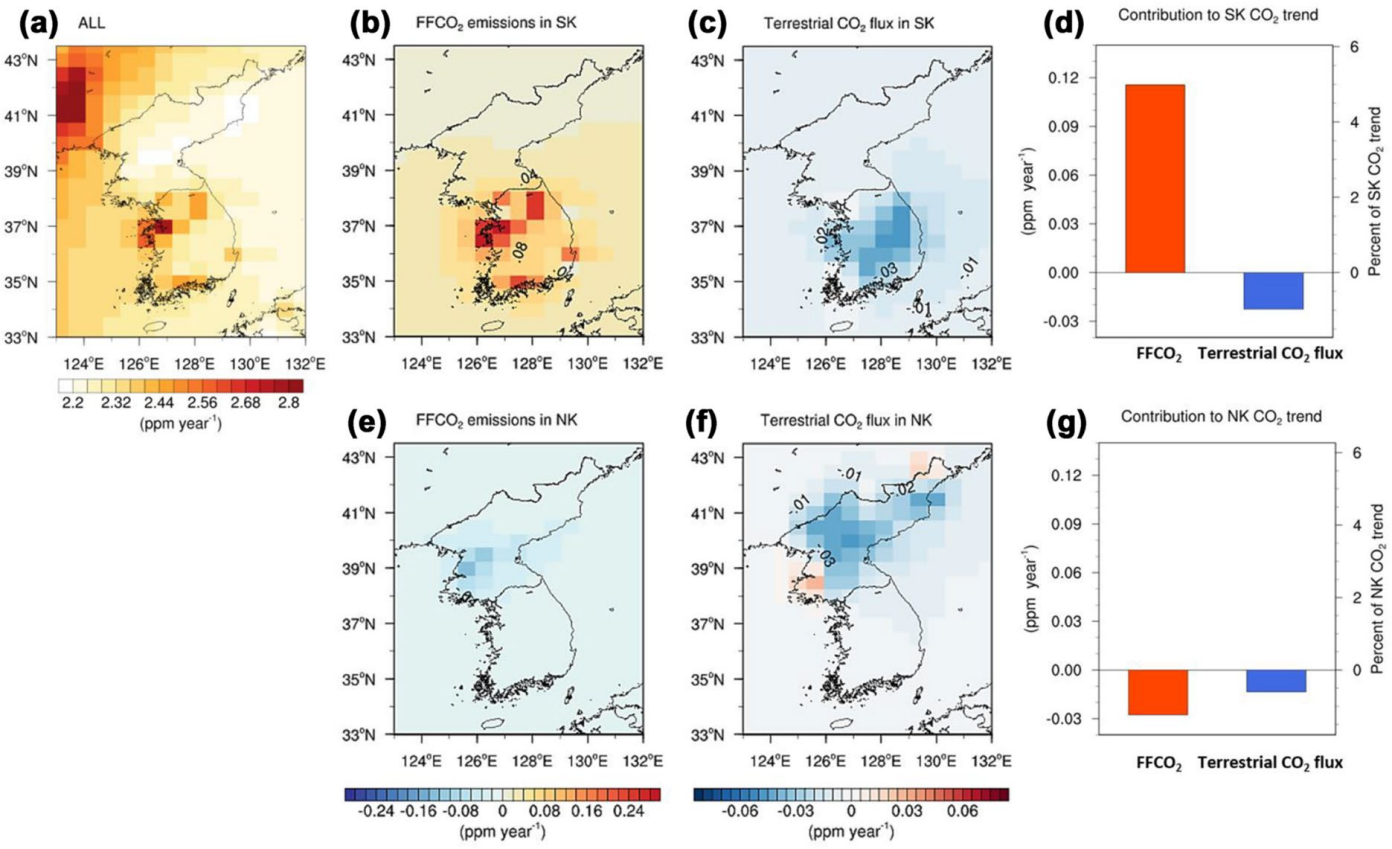
**a** Simulated annual trends of atmospheric CO_2_ from 2000 to 2016 (ALL_transient_); (**b**, **e**) contributions of fossil-fuel CO_2_ (FFCO_2_) emissions [ALL_transient_ minus FF_SK(NK)_2000_]; and (**c**, **f**) terrestrial CO_2_ flux [ALL_transient_ minus BIO_SK(NK)_2000_] in South Korea (SK) and North Korea (NK). (**d**, **g**) Magnitude of contributions at the national scale and their relative contributions to national mean atmospheric CO_2_ trends

**Fig. 4:**
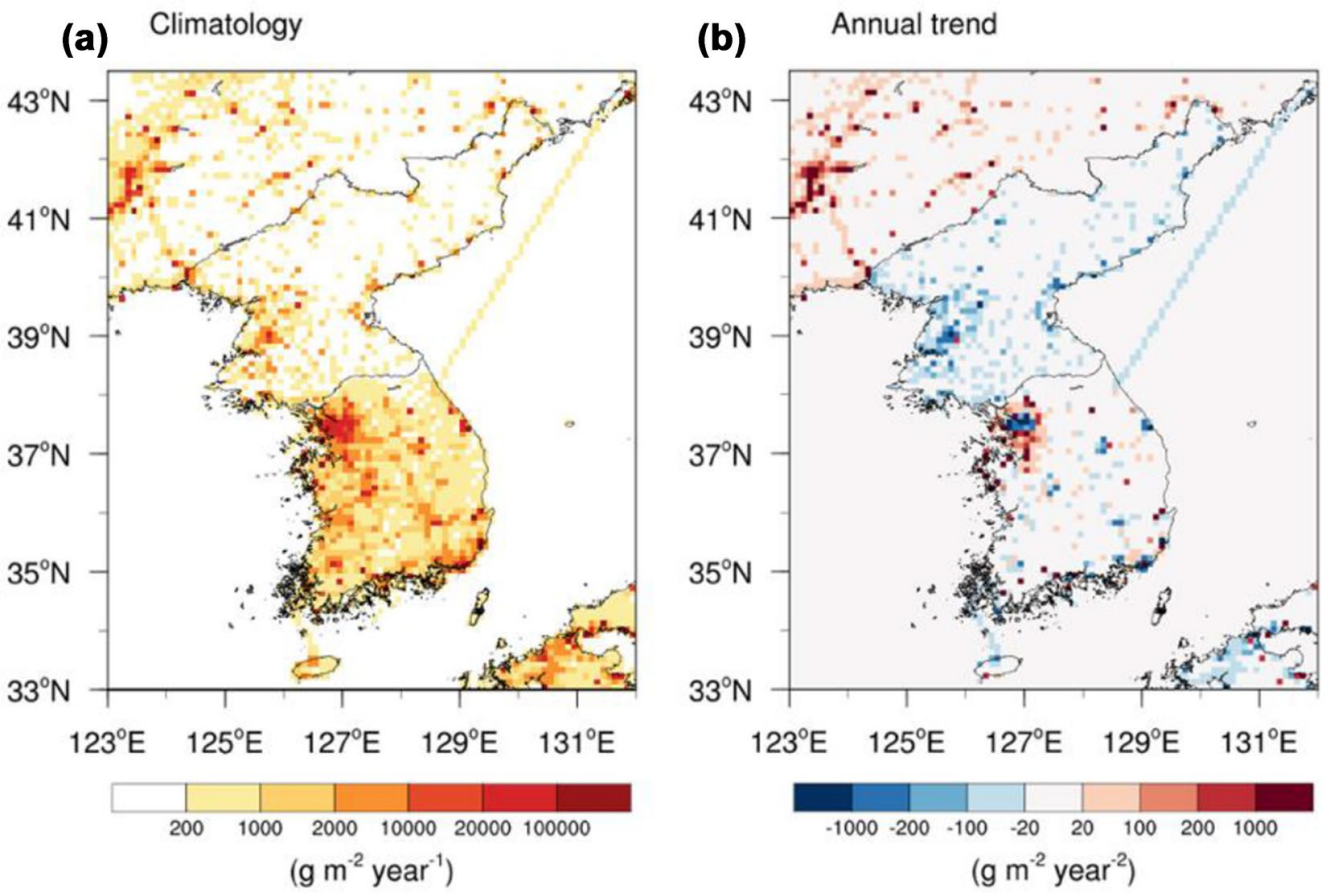
**a** Mean and (**b**) trend of annual fossil-fuel CO_2_ emissions derived from an inventory for the period of 2000–2016. The CO_2_ emissions were computed by summing the emissions from all sectors, including energy, fuel production and use, waste, and transport (ground, shipping, and aviation)

To examine the role of regional CO_2_ flux changes on atmospheric CO_2_ variations over the Korean Peninsula, a set of CTM simulations were conducted for 2000–2016 (details in the “Data and methods” section). In the ALL_transient_ simulation, wherein all variables are transient, the increasing rate of atmospheric CO_2_ is gradually lowering from west to east over the surrounding areas of the Korean Peninsula during 2000–2016 owing to the heavily industrialized provinces (e.g., Liaoning) in northeastern China (Figs. 3a, 4a, b) [27, 28]. In addition, distinctly different spatial patterns of atmospheric CO_2_ trends were present between these countries. Specifically, an increase of more than 2.4 ppm year^− 1^ occurred in the outskirts of Seoul and certain industrial complexes (e.g., Yeosu and Ulsan), located in the northwest and southeast parts of South Korea, respectively. In contrast, the central and northeast regions of North Korea presented a relatively lower increase (2.2 ppm year^− 1^) than the adjacent surrounding areas.

Sensitivity simulations, which evaluate the effect of surface CO_2_ fluxes and atmospheric transport on atmospheric CO_2_ variations, revealed that the increasing FFCO_2_ emissions in South Korea is the major driver of regional differences in atmospheric CO_2_ trends between these countries. In particular, the increase in FFCO_2_ emissions, particularly in major cities, rose the regional and country atmospheric CO_2_ concentrations by more than 0.3 ppm year^− 1^ and 0.12 ppm year^− 1^, respectively, accounting for 5 % of the net increase in national atmospheric CO_2_ (Figs. 3b, d, 4b). The increasing rate of atmospheric CO_2_ was relatively lower in Seoul than in the surrounding areas because emissions in this area have decreased owing to the government’s policy to shift industrial facilities from Seoul to its outskirts (Figs. 3b, 4b). In the northeastern part of South Korea, approximately 0.2 ppm year^− 1^ of the national increase appears to be caused by one strong point source. However, no possible sources of FFCO_2_, such as coal-burning power plants, are present in this mountainous area. Thus, this area may have been mis-allocated; hence, cautious interpretation of the spatial map is necessary. Conversely, in North Korea, decreases in FFCO_2_ emissions, particularly in the Pyongyang metropolitan area, which includes several primary source regions, reduced the CO_2_ concentrations in the region by more than 0.1 ppm year^− 1^, presenting a small nationwide impact of − 0.03 ppm year^− 1^ (Figs. 3e, g, 4b). Moreover, the CT estimates, which are greater than those of the TRENDY multi-model mean, indicate that increases in terrestrial CO_2_ uptake over widely distributed forests in the two countries decreased atmospheric CO_2_ by up to 0.04 ppm year^− 1^ (Fig. 3c, f). Although the nationwide effect of terrestrial uptake in South Korea (–0.02 ppm year^− 1^) is greater than that in North Korea (–0.01 ppm year^− 1^), its magnitude is too small to offset the increase in CO_2_ concentrations induced by increasing FFCO_2_ emissions (Fig. 3d, g).

**Fig. 5:**
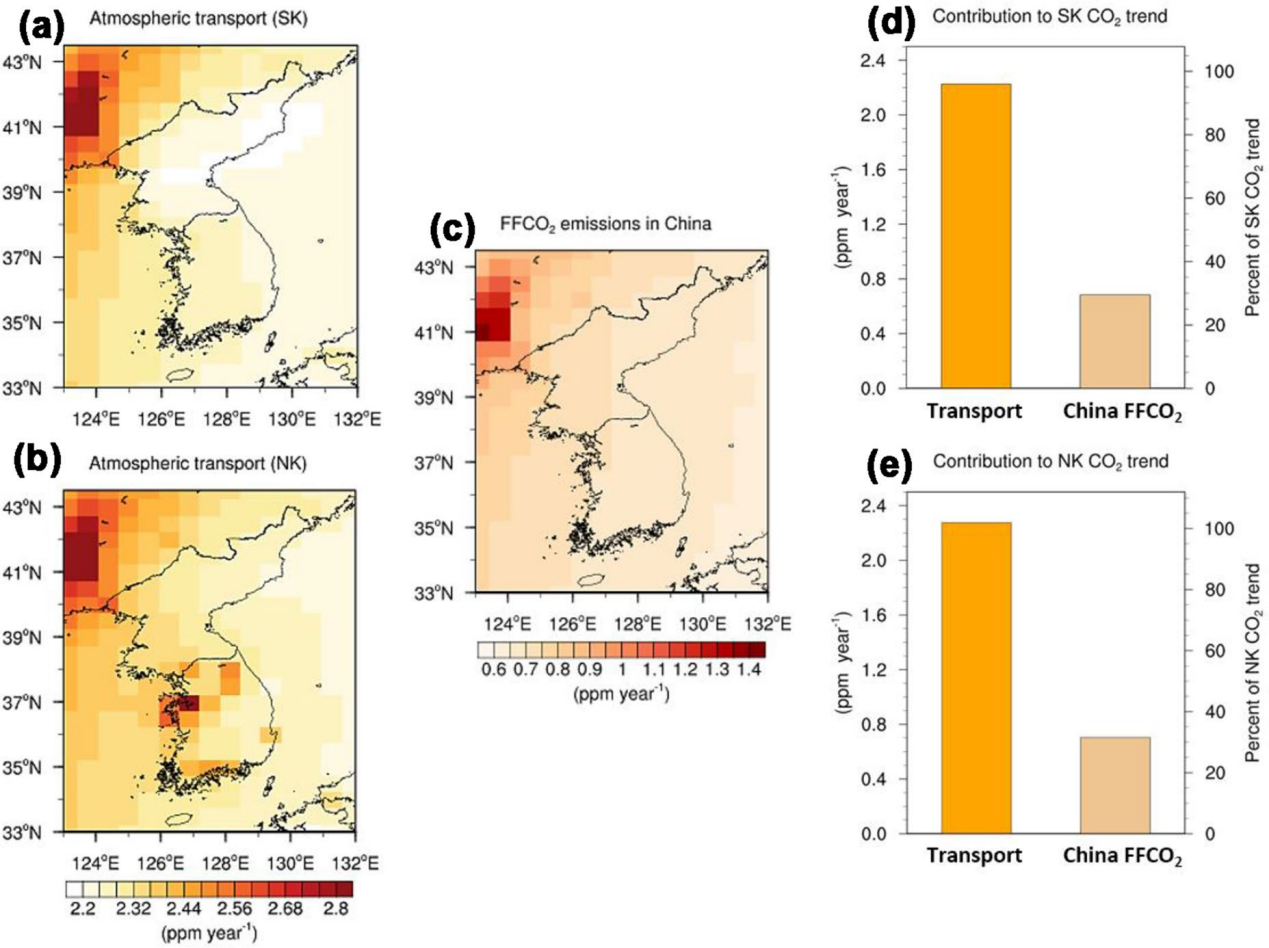
**a–c** Contributions of changes in transported CO_2_ from outside South Korea (SK) (FFBIO_SK; **a**) and outside North Korea (NK) (FFBIO_NK; **b**) and increases in fossil-fuel CO_2_ (FFCO_2_) emissions in China (ALL_transient_ minus FF_CH_2000_; **c**) to simulated annual trends of atmospheric CO_2_ in SK and NK. **d**, **e** Magnitude of contributions at national scale and relative contributions to national mean atmospheric CO_2_ trends

The changes in transported CO_2_ similarly rose the atmospheric CO_2_ concentrations in both South and North Korea by 2.23 ppm year^− 1^ and 2.27 ppm year^− 1^, respectively (Fig. 5d, e). The greater increase in atmospheric CO_2_ in North Korea is the result of its geographic proximity to major carbon sources in northeastern China and South Korea. Although the changes in transported CO_2_ did not cause distinct regional differences in atmospheric CO_2_ trends, they comprise more than 95 % of net increases in atmospheric CO_2_ in these countries. Specifically, the increase in FFCO_2_ emissions in China, accounting for 65 % of the global FFCO_2_ increases in the period as derived from the national FFCO_2_ emission inventory [26], caused South and North Korea to present greater increasing rates of atmospheric CO_2_ than the global mean (0.56 ppm year^− 1^), rising at rates of 0.68 ppm year^− 1^ and 0.70 ppm year^− 1^, respectively (Fig. 5c, d, e). Nevertheless, the contribution of rising FFCO_2_ emissions from China to the increases in atmospheric CO_2_ over the Korean Peninsula is relatively smaller (30–31 %) than the contribution of global FFCO_2_ increases. This is because the atmospheric CO_2_ concentration is bound to increase every year, even if global FFCO_2_ emissions remain at 2000 levels, because FFCO_2_ emissions have been greater than the natural carbon absorption since industrialization [1].

## Discussion

Stronger actions are required in each country to mitigate climate change [29]. To support these efforts, a scientific understanding of the characteristics of national carbon budgets and the response of atmospheric CO_2_ to budget changes is required. This study examined the causes of atmospheric CO_2_ variations at the national level via a case study of the Korean Peninsula. The CTM simulations showed that the increasing rate of atmospheric CO_2_ is greater in South Korea than in North Korea because of the contrasting trends of FFCO_2_ emissions over the last two decades. These contrasting trends correspond to the differing economic growth strategies influencing the energy structure. In particular, North Korea has created a self-reliant national economy based on its forest and mineral resources, especially coal. After the economic collapse in the 1990s, North Korea exported a significant amount of coal to China to revive its economy [30]. Because coal exports increased without the restoration of coal mine damage caused by the Great Flood in the 1990s, the domestic coal consumption and resulting CO_2_ emissions decreased. Conversely, South Korea has experienced successful export-led manufacturing growth since the 1960s [31]. Our results show that the efforts to reduce carbon emissions were already underway via renewable energy supply expansion, as it is common for developed countries to shift toward reducing carbon emissions after achieving a certain economic level [32, 33]. However, the increase in renewable energy was insufficient to suppress the increase in fossil energy use. Moreover, the results indicate that the regional distribution of long-term changes in atmospheric CO_2_ is mainly constrained by the national economic conditions in Korea.

**Fig. 6:**
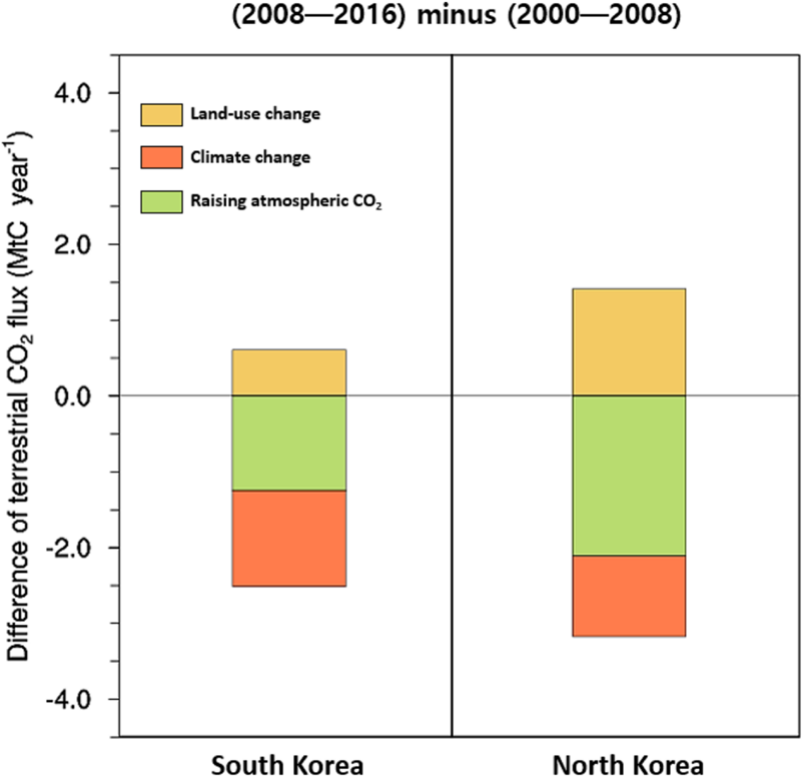
Contributions of rising atmospheric CO_2_ (simulation S1 minus simulation S0), climate change (simulation S2 minus simulation S1), and land-use change (simulation S3 minus simulation S2) to estimate the difference in national terrestrial CO_2_ flux between the periods of 2008–2016 and 2000–2008 in South Korea and North Korea estimated from nine TRENDY multi-model means. Simulation S3 was forced with historical changes in atmospheric CO_2_, climate, and land use. The annual land use did not vary in simulation S2, while the changes in climate and land use were not prescribed in simulation S1. Meanwhile, in simulation S0 was not forced with the annual changes of any of the factors

In addition to the effect of FFCO_2_ emissions, terrestrial ecosystems partially contributed to changes in national atmospheric CO_2_ concentrations. Both inverse modeling and process-based models estimated that terrestrial CO_2_ uptake has not only increased in South Korea but also in North Korea, wherein a notable decrease in forest area has been observed from space [9]. We obtained insights into the causes of increased terrestrial CO_2_ uptake using the TRENDY sensitivity simulation results (details in the “Data and methods” section). Following the land cover change, the TRENDY models estimated that the decrease in terrestrial CO_2_ uptake by land-use change is greater in North Korea than in South Korea (Fig. 6). However, the effects of rising atmospheric CO_2_ and climate change, which have enhanced vegetation growth in mid-to-high latitude regions [34, 35], were greater than the impact of land-use changes. This indicates that terrestrial ecosystems in North Korea, where deforestation is still ongoing, also play a role in alleviating the atmospheric CO_2_ increase, as South Korea, wherein forest management has been conducted for a long time.

The CTM simulations showed that changes in atmospheric transport are a minor factor inducing regional differences in the annual trends of atmospheric CO_2_ concentrations in the Korean Peninsula. Even on a monthly scale, similar variations in atmospheric CO_2_ in the two countries occurred in winter, indicating that they are generally under the effect of the same atmospheric circulation system. However, changes in atmospheric transport play a major role in differentiating the CO_2_ concentrations among these countries and other regions from a global perspective. Specifically, the atmospheric CO_2_ increase rate in North Korea is greater than the global mean value, even though their net carbon emissions have decreased. Previous studies reported that the greatest growth rate of atmospheric CO_2_ has been observed in the Korean Peninsula, as compared with background sites in East Asia [22], especially when wind blows from China [19]. In line with the observation studies, our results show that the increase in FFCO_2_ emissions in China, along with the increase in FFCO_2_ emissions from South Korea, is the major drivers of rising atmospheric CO_2_ concentrations in the Korean Peninsula. These results suggest that the effect of atmospheric transport should be considered when monitoring changes in regional carbon budgets via observational studies, especially when quantifying local CO_2_ enhancement by comparison with other regions (e.g., [17, 21, 23]).

## Conclusions

In this study, we discovered that different economic conditions between South and North Korea led to regional differences in their increasing rates of atmospheric CO_2_ over the last two decades. However, from a global perspective, changes in atmospheric transport are the main factors causing greater increases in atmospheric CO_2_ in these countries, as compared with the global average increase. Our results highlight the importance of accurately separating the influences of atmospheric transport and regional carbon budget changes on atmospheric CO_2_ variations in establishing effective plans to achieve national carbon neutrality. This study, based on CTM simulations and various modeling and statistical datasets, provides directions for interpreting obtained atmospheric CO_2_ data from surface and satellite measurements in relation to national economic structure, terrestrial ecosystems, and atmospheric transport, especially in main source regions. Moreover, our results show that only approximately 5 % of the increase in CO_2_ concentration can be mitigated in South Korea, even if their FFCO_2_ emissions, ranked among the world’s top 10 in 2018 [7], are maintained in 2000. This indicates that the rise in atmospheric CO_2_ cannot be arrested unless all countries around the world achieve carbon neutrality, despite policies for carbon neutrality being implemented individually by each country.

## Data and methods

### Atmospheric CO_2_ measurements

Weekly atmospheric CO_2_ measurements have been conducted globally by the cooperative global air sampling network. The global monthly mean surface atmospheric CO_2_ concentration was computed by the National Oceanic and Atmospheric Administration-Earth System Research Laboratories (NOAA-ESRL) using global atmospheric CO_2_ measurements from 2000 to 2016 [36]. To obtain the global monthly estimate, the weekly CO_2_ measurements at sites less affected by local land sources and sinks were fitted to a smooth curve by applying curve fitting and filtering techniques [37]. We used the data to identify the regional characteristics of atmospheric CO_2_ variations over Korea that are distinct from the global mean changes and evaluate the performance of our atmospheric transport model simulations.

### Statistics of anthropogenic CO_2_ emissions and energy consumption

The Emissions Database for Global Atmospheric Research version 5.0 (EDGAR v5.0; [26]) provides annual anthropogenic CO_2_ emissions from 22 sectors on a per country basis on a 0.1° grid over 1970–2018 (values for 2016–2018 are obtained from fast track methodology) [38]. The dataset includes all FFCO_2_ sources, including fossil fuel combustion, cement production, shipping, and aviation. The EDGAR v5.0 was used to investigate changes in FFCO_2_ emissions in South and North Korea during 2000–2016 and estimate the effects of the emission changes on atmospheric CO_2_ variations from atmospheric transport model simulations. In addition, the annual total primary energy consumption and composition of energy sources were investigated based on national statistics from the Korean Statistical Information Service, to understand the differing FFCO_2_ emission trends between the two countries [39].

### Terrestrial CO_2_ flux

To estimate changes in the terrestrial CO_2_ flux over Korea during 2000–2016, two types of modeling results were used: inverse modeling and process-based models. First, we investigated the monthly averaged terrestrial CO_2_ flux estimated by CT (i.e., CT2017), which uses the global transport model version 5 and atmospheric CO_2_ measurements from 151 surface observation sites, as well as aircraft and shipboard [40]. The CT2017 dataset has the highest spatial resolution (1°) among the widely used and publicly available inversion datasets. We then used the monthly averaged net biome production (NBP) simulated by dynamic global vegetation models involved in the TRENDY project version 6, which follow historical changes in atmospheric CO_2_, climate, and land use (simulation S3; [41]). Next, we calculated the average changes in NBP over the regions and their variance simulated using nine models: CABLE, CLM4.5, ISAM, LPJ, LPX-Bern, ORCHIDEE, VEGAS, VISIT, and JULES. The TRENDY models also provided the results of sensitivity simulations. Simulation S1 was forced with increasing atmospheric CO_2_ and simulation S2 was forced with increasing atmospheric CO_2_ and climate change; meanwhile, simulation S0 was not forced with the annual changes of any of the factors. By comparing the simulations with or without the annual changes for each factor, we estimated the causes of changes in terrestrial CO_2_ fluxes over North and South Korea.

### GEOS-Chem model simulations

Goddard Earth Observing System-Chemistry model (GEOS-Chem) is a CTM that simulates the 3-D field of the atmospheric CO_2_ concentration using datasets for meteorological variables and surface CO_2_ fluxes [42, 43]. We utilized a nested-grid GEOS-Chem model (version 11.2) to estimate the global mean changes in atmospheric CO_2_ and its spatiotemporal variations over Korea. Results from the global simulation with a 4° × 5° horizontal resolution were prescribed as boundary conditions for the nested-grid simulations, which had a 0.5° × 0.625° horizontal resolution and 47 vertical layers over East Asia. All model simulations were conducted using datasets for hourly meteorological variables [44], annual anthropogenic CO_2_ emissions (EDGAR v5.0), monthly terrestrial CO_2_ flux (CT2017), climatological ocean CO_2_ flux [45], and monthly biomass burning [46].

After a 10-year spin-up, a set of sensitivity simulations were conducted for 2000–2016 to evaluate the influences of changes in regional CO_2_ sources and sinks and atmospheric transport on atmospheric CO_2_ spatiotemporal variations over Korea (Table 1). In ALL_transient_, the transient meteorological variables and surface CO_2_ fluxes were applied during the simulation period. In FF_SK_2000_, BIO_SK_2000_, and FFBIO_SK_2000_, one or both of the FFCO_2_ emissions and terrestrial CO_2_ flux in 2000 were repeated in South Korea while the input variable conditions were the same as in ALL_transient_. These three simulations were repeated by switching the area from South Korea to North Korea: FF_NK_2000_, BIO_NK_2000_, and FFBIO_NK_2000_. According to the difference in the simulated surface CO_2_ concentrations between ALL_transient_ and FF_SK_2000_ (FF_NK_2000_) and BIO_SK_2000_ (BIO_NK_2000_), the influences of regional changes in FFCO_2_ emissions and terrestrial CO_2_ flux on atmospheric CO_2_ variations over South Korea (North Korea) were estimated. Further, based on the simulated surface CO_2_ concentrations in FFBIO_SK_2000_ and FFBIO_NK_2000_, the influence of atmospheric transport changes on atmospheric CO_2_ variations over the regions was estimated.

**Table 1:** Model simulation configuration

Simulations	Descriptions		Simulations	Descriptions	
	Fossil-fuel CO_2_ emissions	Terrestrial CO_2_ flux		Fossil-fuel CO_2_ emissions	Terrestrial CO_2_ flux
ALL_transient_	T	T			
FF_SK_2000_	FIX^a^	T	FF_NK_2000_	FIX^b^	T
BIO_SK_2000_	T	FIX^a^	BIO_NK_2000_	T	FIX^b^
FFBIO_SK_2000_	FIX^a^	FIX^a^	FFBIO_NK_2000_	FIX^b^	FIX^b^
FF_CH_2000_	FIX^c^	T			

Set of model simulations employed to estimate the influences of changes in regional land-surface CO_2_ fluxes and atmospheric transport on CO_2_ concentrations over South Korea (SK) and North Korea (NK) for during 2000–2016

T: All variables are transient

FIX^a^, FIX^b^, FIX^c^: CO_2_ flux in 2000 is repeatedly prescribed in SK, NK, and eastern China, respectively

To provide an additional explanation for the effect of atmospheric transport changes, we performed the FF_CH_2000_ simulation, which is the same as the FF_SK_2000_ simulation; however, the FFCO_2_ emissions in 2000 were repeated over eastern China (approximately 20–40°N, 100–125°E and 40–50°N, 100–140°E) during the simulation period. The influence of changes in FFCO_2_ emissions in China on atmospheric CO_2_ variations over Korea were evaluated based on the difference in simulated surface CO_2_ concentrations between ALL_transient_ and FF_CH_2000_. The modeling bias that overestimated the long-term trend of increasing CO_2_ concentration was corrected by comparing the measured CO_2_ concentration at Mauna Loa, which is widely used as a global (or Northern Hemisphere) background site. The bias-corrected GEOS-Chem results show a good match with the NOAA-ESRL flask CO_2_ measurements in South Korea because atmospheric measurements were used to assimilate the terrestrial CO_2_ flux within the CT inversion system (not shown here). The introduced model simulation set was used to identify the causes of changes in monthly variations of atmospheric CO_2_ over South Korea [19].

## Data Availability

The surface atmospheric CO_2_ measurement datasets are available at https://www.esrl.noaa.gov/gmd/dv/data/. The Emissions Database for Global Atmospheric Research v5.0 datasets are publicly available at http://jeodpp.jrc.ec.europa.eu/ftp/jrc-opendata/EDGAR/datasets/v50_GHG/. The TRENDY model simulation results are available from Stephen Sitch (S.A.Sitch@exeter.ac.uk) or Pierre Friedlingstein (p.friedlingstein@exeter.ac.uk) upon email request. The CarbonTracker results are publicly available at http://carbontracker.noaa.gov. The datasets of primary energy supply in South Korea and North Korea are available at https://kosis.kr/statHtml/statHtml.do?orgId=101&tblId=DT_1ZGA72 (in Korean). The GEOS-Chem model simulation results that support the findings of this study are available from S.J. upon request.

## Abbreviations

UNFCCC: United Nations Framework Convention on Climate Change
GDP: Gross domestic product
FFCO_2_: Fossil-fuel CO_2_
CTM: Chemical transport model
GEOS-Chem: Goddard Earth Observing System-Chemistry model
Mtoe: Million tons of oil equivalent
NBP: Net biome production
EDGAR: Emissions Database for Global Atmospheric Research
CT: CarbonTracker
NOAA-ESRL: National Oceanic and Atmospheric Administration-Earth System Research Laboratories
